# Addressing complex pharmacy consultations: methods used to develop a person-centred intervention to highlight alcohol within pharmacist reviews of medications

**DOI:** 10.1186/s13722-021-00271-5

**Published:** 2021-10-16

**Authors:** Jim McCambridge, Karl Atkin, Ranjita Dhital, Brent Foster, Brendan Gough, Mary Madden, Stephanie Morris, Ronan O’Carroll, Margaret Ogden, Anne Van Dongen, Sue White, Cate Whittlesea, Duncan Stewart

**Affiliations:** 1 Department of Health Sciences, University of York, Seebohm Rowntree Building, Heslington, YO10 5DD York UK; 2 Department of Pharmacy, University of Reading, Reading, UK; 3 Whitworths Chemists, Middlesbrough, UK; 4 School of Social Sciences, Leeds Beckett University, Leeds, UK; 5 Department of Psychology, Stirling University, Stirling, UK; 6 North of England Commissioning Support (NECS), Newcastle, UK; 7 School of Pharmacy, University College London, London, UK

**Keywords:** Alcohol, Complex interventions, Pharmacist, Brief intervention, Person-centred, Medications review

## Abstract

**Background:**

Alcohol is challenging to discuss, and patients may be reluctant to disclose drinking partly because of concern about being judged. This report presents an overview of the development of a medications review intervention co-produced with the pharmacy profession and with patients, which breaks new ground by seeking to give appropriate attention to alcohol within these consultations.

**Methods:**

This intervention was developed in a series of stages and refined through conceptual discussion, literature review, observational and interview studies, and consultations with advisory groups. In this study we reflect on this process, paying particular attention to the methods used, where lessons may inform innovations in other complex clinical consultations.

**Results:**

Early work with patients and pharmacists infused the entire process with a heightened sense of the complexity of consultations in everyday practice, prompting careful deliberation on the implications for intervention development. This required the research team to be highly responsive to both co-production inputs and data gathered in formally conducted studies, and to be committed to working through the implications for intervention design. The intervention thus evolved significantly over time, with the greatest transformations resulting from patient and pharmacist co-design workshops in the second stage of the process, where pharmacists elaborated on the nature of the need for training in particular. The original research plans provided a helpful structure, and unanticipated issues for investigation emerged throughout the process. This underscored the need to engage dynamically with changing contexts and contents and to avoid rigid adherence to any early prescribed plan.

**Conclusions:**

Alcohol interventions are complex and require careful developmental research. This can be a messy enterprise, which can nonetheless shed new insights into the challenges involved in optimising interventions, and how to meet them, if embraced with an attitude of openness to learning. We found that exposing our own research plans to scrutiny resulted in changes to the intervention design that gained the confidence of different stakeholders. Our understanding of the methods used, and their consequences, may be bounded by the person-centred nature of this particular intervention.

## Background

Alcohol damages health, being a cause of more than 200 diseases, injuries and other health and social problems; it is responsible for almost 5% of deaths worldwide, and more than twice that proportion in Europe [1]. The Global Burden of Disease study found that drinking alcohol was the leading cause of death in 2016 for the world’s population aged 15–49 years, accounting for approximately 8% of all deaths [2]. Among those aged 50 or older, cancers accounted for a large proportion of total alcohol-attributable deaths (approximately 27% in women and 19% in men). Health systems across the world are contending with the challenges posed by multimorbidity as populations age, with alcohol deeply implicated in ways we are only now beginning to fully understand [3]. Alcohol costs society much more than tobacco in purely monetary terms, perhaps twice as much [4, 5], as well as being responsible for unquantified levels of suffering to drinkers, and to those around them.

Paradoxically, despite the widespread nature of such risks and harms, one’s own alcohol consumption is not an easy subject for either patients or practitioners to discuss within healthcare services [6]. Most studies have involved general practitioners where time constraints and dealing with multiple connected issues in consultations, uncertainties about roles and how to help, the particular skills needed, and discomfort related to practitioners’ own drinking are all relevant to how such discussions unfold [7, 8].

Alcohol is deeply embedded in personal, family and community lives, including those of both patients and practitioners, and their views on celebration, relaxation, inhibition, and socialising. More than 30 years ago the Royal College of Psychiatrists published *Alcohol: our favourite drug* [9], yet alcohol is rarely clearly identified as a drug in contemporary policy, public, health promotion and clinical discourses. Alcohol’s addictive nature and neurotoxic effects on the brain leaves no doubt that it is a drug; indeed alcohol is the world’s favourite drug [10].

Alcohol can cause harm at low levels of consumption and there is no entirely safe dose [2]. Patterns of drinking that seem unremarkable, neither being very frequent nor involving consumption of large quantities, are likely to cause harm when there are existing health problems, and particularly so if there are multiple chronic conditions [3]. Alcohol industry bodies emphasise ‘responsible drinking’ of their products, focusing on the individual user and deflecting attention from their own roles in the production of the harms [11, 12]. This is similar to how the tobacco and gambling industries emphasise the rights and responsibilities of consumers using their products [13, 14]. This deflecting construction is also found in widespread injunctions from policymakers on ‘responsible drinking’ [5]. Similarly vague notions such as “moderate” drinking that eschew any identification of precise doses, or their possible effects, are also widespread. Alcohol dose matters greatly in how the drug affects the body, especially for people with long term conditions treated with multiple medications.

Such constructs do not assist people to think meaningfully and precisely about their own drinking, or its consequences, or to discuss it with someone else as a way to clarify one’s thinking. In countries such as England that rely on industry organisations for public education [15], this may be especially challenging. Sophisticated marketing campaigns shape perceptions of alcohol in positive ways [16–18], from childhood onwards [19], and alcohol is central to the social media presentation of young adult identities [20]. It is therefore unsurprising that alcohol is not an easy subject for patients and practitioners to discuss with each other.

For almost 40 years the research literature on how to raise the subject of alcohol and then discuss it has developed based on a paradigm established in a World Health Organization sponsored series of studies on screening and brief interventions in general practice [21–24]. This paradigm is somewhat dissolving as leading proponents of this approach seek new intervention models [8, 25–27], and despite early optimism, no longer regard any population health benefits as likely in the absence of wider supportive policy measures [28]. The limited strength of existing evidence of effectiveness in routine healthcare settings [8] is striking given the likely scale of the burden on health services, which appears underappreciated [3]. Implementation of screening and brief intervention programmes is proceeding in many countries and may help alcohol and health to be more widely discussed. It may also have unintended consequences. The nature of screening is prominent among the uncertainties discussed in the research community, and it has been proposed that there is a “need to find smarter ways to initiate discussions about alcohol” [29].

Policy developments in many countries promote the potential public health function of community pharmacies, which presents an opportunity to extend the reach of brief interventions. A trial in London, however, demonstrated no evidence of benefit [30], and a nested qualitative study identified that there was missing proof of concept, as participants who were risky drinkers saw no need for intervention; they held stereotypical ideas about the nature of alcohol problems, and their drinking did not correspond with the stereotype [31].

Rather than asking pharmacists to take on a new public health role in delivering standalone brief interventions targeting only alcohol, we have developed an intervention approach which integrates attention to alcohol within what pharmacists themselves may recognise as good professional practice, and which can be incorporated within routine service delivery. Alcohol is a drug implicated in medication adherence, safety and effectiveness issues for many patients and is thus a legitimate subject for discussion in medicines reviews. Recognition that alcohol itself is not pharmacologically inert challenges current ideas and practice positioning drinking simply as a lifestyle issue separated from medication issues [32]. We sought to develop a novel clinical intervention that includes alcohol within medications reviews for people using multiple medications for chronic conditions who drink alcohol twice a week or more frequently [33]. This study provides an overview and critical reflection of the methods used.

## Methods

### An overview of the research process

**Fig. 1:**
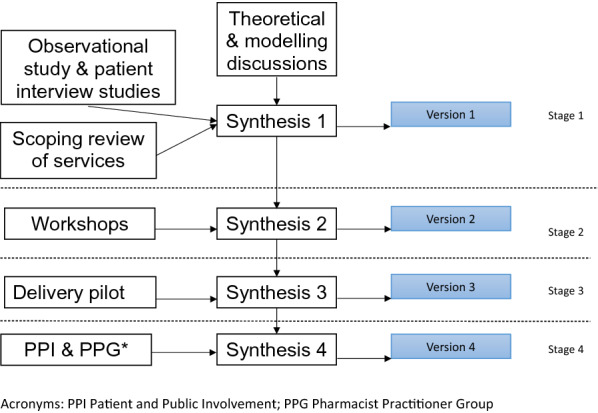
Overview of the 4 stages of the process

The programme of study started from the premise that the intervention should be co-produced with the pharmacy profession and with patients. In our funding application to the U.K. National Institute of Health Research, we identified a range of intervention development, feasibility and acceptability studies necessary to prepare the intervention and trial design for a definitive evaluation study. The pre-trial phase comprised a series of stages; corresponding to the production of versions of the intervention as set out in Fig. 1. A patient interview study was undertaken with 25 patients drinking twice a week or more frequently and taking medications for long term conditions [34]. An exploratory ethnographic observational study examined routine practice among 9 practitioners in 5 pharmacies, observing 31 consultations [32]. A scoping review of the literature on the particular services being studied [35] also preceded the development of Version 1 of the intervention. Then, separate co-design workshops with 14 patients and 7 pharmacists were arranged to examine Version 1 content. The intervention development team studied the data from the workshops, and synthesised it with earlier work, yielding revisions that redefined the intervention in Version 2. A study of how Version 2 of the intervention was conducted in practice resulted in Version 3, again following data synthesis by the team. Consultation with the research programme patient and pharmacist advisory groups similarly led to the production of Version 4. In parallel, in stages 1 and 4 we convened theoretical and modelling discussions within the research team to provide a context for the integration of the empirical research strands. Preparation for each stage of the research involved detailed intervention development team discussions. The planned work was completed on schedule after 15 months, though we found that during the course of intervention development the various changes to the character and detailed content of the intervention generated new research needs. We thus embarked on a planned pilot trial undertaken in 10 community pharmacies after Stage 4, also intending to do further intervention development work to produce an initially unplanned finalised Version 5.

### The theoretical approach taken to the intervention development process

There are different views about complex interventions. In the original Medical Research Council guidance [36], complexity is an intrinsic property of interventions, which can be characterized in multiple ways, including the number and difficulty of the behaviours required by those delivering or receiving the intervention; the nature of the target groups and the range of possible effects; and the degree of flexibility or tailoring of the intervention permitted. Alternatively, interventions may be conceptualised as events in complex adaptive systems [37], and these events may themselves be simple or complex, directing attention towards contextual and environmental influences. A third perspective argues that “there are no ‘simple’ or ‘complex’ interventions, and that simplicity and complexity are instead pragmatic perspectives adopted by researchers to help describe and understand the interventions in question” [38]. Our thinking was much more in line with these latter two perspectives than the first, in keeping with a “bottom-up” data-led approach to co-production.

We understood that patients who participate in medication reviews hold their own ideas about their conditions, treatment, and about alcohol. We conceptualised drinking as posing a potential problem directly via its impact on health and well-being, and indirectly by potentially reducing adherence to, or the safety and effectiveness of, medications. We drew on Leventhal’s Common-Sense Model of self-regulation [39], and the necessity/concerns framework [40], and were pragmatic and inductive in selecting content from different theories that fitted the qualitative data.

### The present study: how we have sought to learn from the intervention development process

This study pools data from the intervention development team discussions to produce an account of how the design of the intervention developed over time [41], identifying the key considerations that informed the decision-making. It will be seen that the intervention evolved in fundamental ways. We then reflect on the process, with a view to identifying transferable findings on the intervention development methods used. As we have published a number of the component studies, we take a different approach to the comprehensive account provided by Gaume and colleagues [42], in which all data is included within a single exhaustive report.

## Results

### How the intervention development process unfolded during Stage 1

The ethnographic observation study provided essential grounding in the day-to-day realities of community pharmacy service provision, and deepened our understanding of the nature of the many challenges involved in integrating alcohol into medication discussions [32]. The interview study provided proof of concept that patients who drank alcohol regularly were open to the idea of a discussion that linked alcohol to medications if this was well designed [34]. Many, however, thought that this was not very relevant to them because they did not regard their drinking as problematic, so we further explored risk perceptions [43]. A further analysis of the interview data examined aspects of how patients actually talked about drinking [44]. These studies further developed our appreciation of the complexities inherent in conversations about drinking. In parallel we found in our scoping review of medication review services, along with the observational work, that medication review services were not very person-centred, with attention to alcohol consumption largely absent [45]. Although there was much patient-centred rhetoric, this did not extend far into practice in any real depth.

We started by planning to adapt existing pharmacist training, consultation skills models and person-centred practice guidelines, by highlighting alcohol. We sought to condense relevant content to make it user friendly for the pharmacist and produced a brief paper summary guide to how the medications review consultation could be conceptualised in a person-centred way to include alcohol, placing the main preparatory content online. Version 1 content gave attention to existing consultation skills and practice, and to alcohol and medication, role-related material and enhanced consultation skills exercises including scenarios for managing more complex cases and for continued professional development. The extensive material we developed in this stage was shared with our patient and pharmacy advisory groups and the feedback on content was positive and helpful.

The key challenge we knew we faced was how to bridge the gulf between everyday practice and largely online continuing professional development training provision which espoused commitment to patient-centred ideas, but had clear limitations in supporting actual skill acquisition. We also altered the primary intervention aims during Stage 1; no longer specifically helping people to reduce their drinking and the associated risk per se, but now being fundamentally concerned with the relationships between alcohol, the medications people were using and the conditions for which they were being prescribed in the Medicines and Alcohol Consultation (MAC).

### Intervention development in stages 2–4

The co-design workshops were intended to examine the content of Version 1, with one large patient workshop (including one carer/caregiver), co-facilitated by patient advisory group leads, particularly useful for examining how consultations might begin, and the idea of alcohol as a drug that is appropriate for medications review discussions [46].

Two smaller events for pharmacists in different geographical areas explored the training content in depth. It was emphasized that the MAC presented a clear contrast to existing practice and there was willingness to embrace this, though time pressures were concerning, particularly in busier pharmacies. It was recommended that the online content should be condensed as it was anticipated to be used in practitioners’ own time, and thus the capacity of pharmacists to engage deeply with such material was questioned, and additional in-person components were recommended.

The synthesis following the workshops combined the various strands of earlier evidence available to us and these two new data sources. There were not any formal criteria in this or later stages of decision-making. The limitations of our own research plans were exposed in being open and responsive to both patients and practitioners. Our guiding principles involved being committed to understanding the complexities and the challenges, and to seeking to address them as far as possible within our own constraints. We were thus highly pragmatic, with the team shifting our sense of what was possible based on our emerging findings, with the basis of our decision-making at each stage always documented. This could then be explained to stakeholders in ways which gained their confidence.

**Fig. 2:**
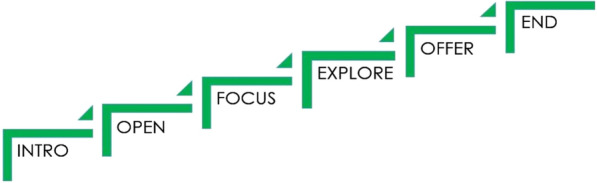
The MAC steps

Version 2 of the MAC comprised a multi-component programme designed to achieve the consultation skills development needed to discuss alcohol in a person-centred manner within medications reviews. This involved two training days implemented to underpin, and integrate with, the other MAC programme elements. The MAC Guide offered a simple steps structure which summarised how the pharmacist could flexibly organise the consultation to be responsive to patient agendas and explore possible connections between alcohol consumption, medicine use and health (see Fig. 2). Practice development outcomes were set (see Table 1).

**Table 1:** Medicines and Alcohol Consultation (MAC) programme practice development outcomes

At the conclusion of the practice development programme, we are aiming for practitioners to: 1.Have developed deeper person-centred consultation skills including through proficient use of counselling microskills and engagement with the MAC steps 2.Be able to use person-centred consultation skills in routine practice to support patients in making use of services provided to benefit their health 3.Be able to integrate an appropriate degree of attention to alcohol within consultations 4.Regard it as good pharmacy consultation practice to explore medications use, conditions and alcohol in a person-centred way 5.Value medication services as providing important opportunities to help patients manage their chronic conditions, and derive the optimal benefits from medications prescribed 6.Have changed consultation practice away from being a quick check of narrowly medication-related issues so that it is not an information-dominated process 7. Manage consultations efficiently and flexibly using the structure provided by the MAC steps 8. Be able to recognise challenging issues in practice, identifying needs for skills development, and formulate plans to address them in the context of CPD 9. Be confident that they are developing patient-centred consultation skills and that further close attention to practice, with support, will develop them further 10. Be committed to further developing patient-centred consultation skills, including using Continuing Professional Development opportunities	

In stage 3 we delivered an abbreviated version of the practice development programme and studied how practitioners used the MAC Guide, support calls, learning resources and buddying opportunities. The MAC Guide comprised a condensed version of key messages from the first training day intended to support practice early on, as practitioners became more accustomed to the approach. As might be expected, there was variability in how practitioners engaged with the MAC programme and delivered the MAC in practice, together with the demands of the research. As well as providing data on feasibility within routine practice, there were also indications of effects in some cases. Testimonies from practitioners about altered consultation practice delivered in interviews were triangulated with interviews with those patients, and in some cases with audio-recordings. This work provided helpful suggestions for further, more modest content development in Version 3.

In Stage 4 we revisited the theoretical and modelling work, aiming to clearly describe the MAC intervention components, to state causal assumptions about how the MAC would work, taking account of contextual factors, and thereby constructing the programme theory to enable faithful delivery and future replication [47]. At this point we used the Theoretical Domains Framework [48] to check the theoretical content breadth and made minor content adjustments having identified no major omissions of relevant theoretical constructs. Because the intervention had evolved more than anticipated during the course of its development, we decided that a process study was needed during the pilot trial in order to finalise the intervention and its underpinning theory. We thus agreed Version 4 with the patient and pharmacy advisory groups, thus completing the planned intervention development process and proceeded with the pilot trial [49], which provided encouraging evidence of impacts, whilst identifying also the challenges involved in research participation for community pharmacists [50]. After we had completed the intervention development work we also examined in depth our experience of the nature of co-production with patients in the context of the relevant literature [51] and further analysed the nature of professionalism among pharmacists in respect of public health roles [52].

### The MAC programme

**Fig. 3:**
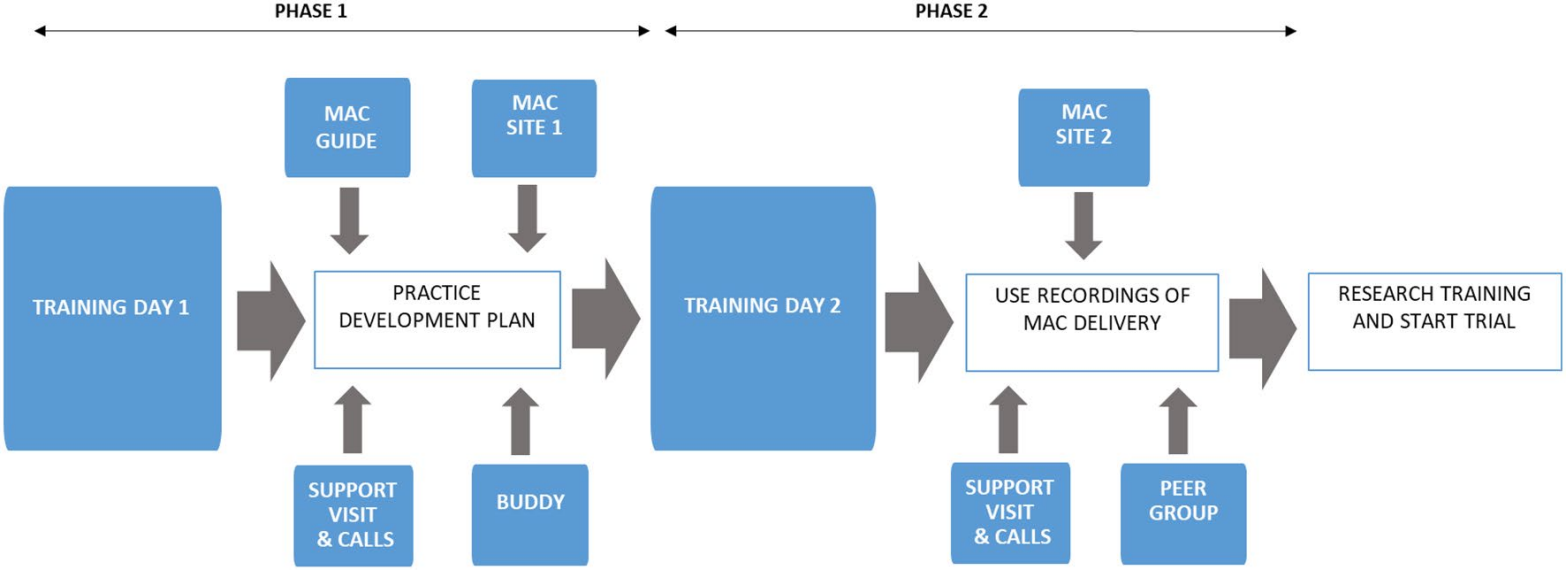
MAC programme overview

Version 4 of the MAC programme comprised eight weeks of training and practice development support and is summarised in Fig. 3. The first training day used interactive sessions with patients, and a focus on core person-centred consultation skills, particularly open questions, and was supported by the additional components identified in Fig. 3 in phase 1 of the programme. There was distinct information provided in the MAC site content in phases 1 and 2, after the practitioners had completed the second training day. That was scheduled four weeks after the first workshop, and focussed on the key issues identified in early use of the MAC in practice, as well as more advanced person-centred skills such as reflective listening and case studies of challenging issues. Throughout, individually tailored practice development support site visits and telephone calls were offered by the MAC support team on a weekly basis, including detailed feedback on audio-recorded MAC consultations after training day 2 (with patient consent). We found audio-recordings particularly valuable to discussions of evolving practice development issues, enabling a focus on specific technical issues for the practitioners, as well as higher level reflection on patient activity in the consultations. We decided not to intervene in informal processes of buddying and peer group support, beyond encouraging these ideas within the training workshops, as we were curious about how this approach might work in the pilot trial. Little such activity resulted, and the lesson was drawn that these components need to be facilitated if retained. The specific skills required to integrate alcohol into consultations became less elusive to pharmacists when examined within recordings of their own practice, which exposed the dynamics of interactions. There were no formalised evaluations of individual practitioner’s skills, with an open discussion about readiness of practice for the trial in light of the Table 1 outcomes preferred instead.

## Discussion

Our intervention was fundamentally transformed in using the methods we set out at the grant application stage. Presenting an account of intervention development in this way offers an opportunity to appreciate potential synergies between individual components, and thus how a complex intervention has been assembled on the basis of a series of planned research studies and other methods. We completed the work described here within the originally planned 15 months, conscious that there was further work to do, and as identified in the subsequent pilot trial, which also addressed RCT feasibility issues. Developing the programme theory is an ongoing process, and major areas of uncertainty, as yet unresolved, include further adaptations to better meet the needs of sub-groups of patients and practitioners. Findings from qualitative process study in the pilot trial will inform this and further intervention design.

Our developmental approach contrasts in a number of informative ways with the methods used by Gaume and colleagues [42], though both involved an iterative qualitative design being applied to the development of brief alcohol intervention, albeit with different populations in different health services contexts. Gaume and colleagues [42] rooted their thinking much more firmly in the existing brief interventions literature, involved external experts (including the first author here) in making decisions, and used formal consensus development methods. Our approach has drawn more extensively on primary qualitative studies and co-production with patients and practitioners. We also rested decision-making authority within the research team (which includes both patients and pharmacists). Interestingly, quite different interventions have resulted from the two programmes of work, and with further research it will be possible to progress brief alcohol intervention development methods. These need to address common challenges intrinsic to the activity of seizing opportunities to explore alcohol’s role in reasons for presentation in different clinical contexts [24].

The experience of the pilot trial has been illuminating for the research team and the pharmacists taking part. Pharmacists were more confident in raising alcohol within medications reviews and had received no negative feedback from patients when doing so. They were also more able to use certain person-centred consultation skills in routine practice to varying degrees. Long standing professional habits and the busy practice context, however, incentivised reverting to more transactional and less person-centred practice. All found the change required sustained effort, which some embraced.

This moment also offers the opportunity to reflect on the research process involved in the development of this complex intervention. Each stage in the process has been valuable, sometimes in quite distinct ways, and being responsive to study findings and guidance from our partners in co-production has meant that the experience has been both rewarding, enlightening and challenging for the researchers involved. As researchers, we have learned in ways that we could not have anticipated at the outset, and the intervention design and content has changed in an iterative process. It has at different times been quite demanding and messy, sometimes in a creative way. Importantly, the structure provided by the research plans and the associated timescales have provided a basis for progress. One aspect to emphasise is the cyclical nature of the developmental process—the more the intervention is developed, the more the scope for new issues to be considered, perhaps until a point of saturation has been reached, which has not yet been achieved.

Achieving a shared understanding of the nature of the issues involved in developing practice takes place within an evolving healthcare system. Changes to NHS contracting arrangements during the pilot trial involved the phasing out of Medicine Use Reviews in community pharmacies. Instead a medications review service has been introduced in general practice, within new organisational structures known as Primary Care Networks [53]. The intervention we have developed has been centred on the pharmacist undertaking medications reviews and will be transportable to this different context, albeit with further preparatory study and refinement needed. Indeed the new service with access to the electronic health record and a more clinical orientation to the patient consultation provides an enhanced platform for person-centred communication, and thus a context beneficial to consideration of alcohol and medications.

We now have an intervention that has been carefully developed that may be useful to both pharmacists and patients. The work already done makes a substantial contribution to the call for rethinking of brief interventions for alcohol [8, 26], by better locating alcohol within the setting and service provision which the patient accesses, and thereby eschewing standalone decontextualized efforts to address drinking. In so doing, this makes a contribution to the forging of a new paradigm to better address the difficult and growing burdens of alcohol and multimorbidity on the NHS and other health systems, partly by finding relatively simple ways to navigate complexity. We know much more now about how pharmacists might talk about alcohol when trying to help people protect their own health, and it is likely that the lessons learned from this experience are transferable to other healthcare professions and settings.

There are obvious study limitations of different kinds. In examining our own processes we may not be best placed to identify the flaws. This study makes a contribution, however, to the wider field of complex intervention development studies, where the emerging norm is to report on one’s own process [41]. There may be much more effective approaches to intervention development, and the MAC may yet not prove to be effective in routine practice, but it is not possible to know this now. After completing this study, we found that many of the lessons we drew from our experience resonated strongly with the messages from the wider field that have found their way into recent guidance [54]. Similarly, there are issues affecting creativity in intervention development arising out of research funding structural constraints seen in the wider literature [55] and it is difficult to identify ‘success’ in intervention development in advance of a trial.

## Conclusions

Alcohol issues are challenging to raise and address in all settings. Complex intervention development research can be a messy enterprise, which can nonetheless shed new insights into the challenges involved in optimising interventions, and how to meet them. Existing guidance provides a useful structure within which to organise research activities focused on understanding the practice, practitioner and patient contexts for intervention design. We found that exposing our own research plans to scrutiny resulted in changes to the intervention design that gained the confidence of different stakeholders. This study may be particularly important to those developing brief interventions, and be of interest also to those studying alcohol and/or pharmacy practice. This study is also highly relevant to wider complex intervention development research, which may benefit from reports of the process as well as the outcomes of existing research programmes.

## Data Availability

Not applicable.

## Abbreviations

MAC: Medicines and Alcohol Consultation
NHS: National Health Service

## References

[1] World Health Organization (2018). Global status report on alcohol and health 2018.

[2] GBD (2016). Alcohol Collaborators: Alcohol use and burden for 195 countries and territories, 1990–2016: a systematic analysis for the Global Burden of Disease Study 2016. Lancet.

[3] Stewart D, McCambridge J (2019). Alcohol complicates multimorbidity in older adults. BMJ.

[4] Towards a smokefree generation: a tobacco control plan for England.

[5] Secretary of State for the Home Department: The Government's Alcohol Strategy. Cm 8336 http://www.homeoffice.gov.uk/publications/alcohol-drugs/alcohol/alcohol-strategy?view=Binary. In*.* London: TSO (The Stationery Office) 2012.

[6] Thom B, Tellez C (1986). A difficult business: detecting and managing alcohol problems in general practice. Br J Addict.

[7] Kaner E, Rapley T, May C (2006). Seeing through the glass darkly? A qualitative exploration of GPs' drinking and their alcohol intervention practices. Fam Pract.

[8] McCambridge J, Saitz R (2017). Rethinking brief interventions for alcohol in general practice. BMJ.

[9] Royal College of Psychiatrists (1986). Alcohol: our favourite drug.

[10] Edwards G (2000). Alcohol: The World's Favourite Drug.

[11] McCambridge J (2012). Dealing responsibly with the alcohol industry in London. Alcohol Alcohol.

[12] Room R (2011). Addiction and personal responsibility as solutions to the contradictions of neoliberal consumerism. Crit Public Health.

[13] Reith G (2007). Gambling and the contradictions of consumption—a genealogy of the "Pathological" subject. Am Behav Sci.

[14] Reith G (2019). Addictive consumption: capitalism, modernity and excess.

[15] McCambridge J, Kypri K, Miller P, Hawkins B, Hastings G (2014). Be aware of Drinkaware. Addiction.

[16] Moodie R, Stuckler D, Monteiro C, Sheron N, Neal B, Thamarangsi T, Lincoln P, Casswell S (2013). Lancet NCDAG: Profits and pandemics: prevention of harmful effects of tobacco, alcohol, and ultra-processed food and drink industries. Lancet.

[17] Hastings G (2013). The marketing matrix: how the corporation gets its power and how we can reclaim it.

[18] Madden M, McCambridge J (2021). Alcohol marketing versus public health: David and Goliath?. Glob Health.

[19] Hastings G, Sheron N (2013). Alcohol marketing: grooming the next generation: children are more exposed than adults and need much stronger protection. BMJ.

[20] Lennox J, Emslie C, Sweeting H, Lyons A (2018). The role of alcohol in constructing gender & class identities among young women in the age of social media. Int J Drug Policy.

[21] Babor TF, Ritson EB, Hodgson RJ (1986). Alcohol related problems in the primary health care setting: a review of early intervention strategies. Br J Addict.

[22] Saunders JB, Aasland OG, Babor TF, de la Fuente JR, Grant M (1993). Development of the Alcohol Use Disorders Identification Test (AUDIT): WHO collaborative project on early detection of persons with harmful alcohol consumption - II. Addiction.

[23] WHO Brief Intervention Study Group (1996). A cross-national trial of brief interventions with heavy drinkers. Am J Public Health.

[24] McCambridge J, Cunningham JA (2014). The early history of ideas on brief interventions for alcohol. Addiction.

[25] Rehm J, Anderson P, Manthey J, Shield KD, Struzzo P, Wojnar M, Gual A (2015). Alcohol use disorders in primary health care: what do we know and where do we go?. Alcohol Alcohol.

[26] Glass JE, Andreasson S, Bradley KA, Finn SW, Williams EC, Bakshi AS, Gual A, Heather N, Sainz MT, Benegal V (2017). Rethinking alcohol interventions in health care: a thematic meeting of the International Network on Brief Interventions for Alcohol & Other Drugs (INEBRIA). Addict Sci Clin Pract.

[27] McCambridge J, Rollnick S (2014). Should brief interventions in primary care address alcohol problems more strongly?. Addiction.

[28] Heather N (2012). Can screening and brief intervention lead to population-level reductions in alcohol-related harm?. Addict Sci Clin Pract.

[29] Andreasson SB (2017). Tackling alcohol use: screening, target group, and patient centred care. BMJ.

[30] Dhital R, Norman I, Whittlesea C, Murrells T, McCambridge J (2015). The effectiveness of brief alcohol interventions delivered by community pharmacists: randomised controlled trial. Addiction.

[31] Quirk A, MacNeil V, Dhital R, Whittlesea C, Norman I, McCambridge J (2016). Qualitative process study of community pharmacist brief alcohol intervention effectiveness trial: Can research participation effects explain a null finding?. Drug Alcohol Depend.

[32] Morris S, Madden M, Gough B, Atkin K, McCambridge J (2019). Missing in action: Insights from an exploratory ethnographic observation study of alcohol in everyday UK community pharmacy practice. Drug Alcohol Rev.

[33] Stewart D, Hewitt C, McCambridge J (2020). Exploratory validation study of the individual AUDIT-C items among older people. Alcohol Alcohol.

[34] Madden M, Morris S, Atkin K, Gough B, McCambridge J (2019). Patient perspectives on discussing alcohol as part of medicines review in community pharmacies. Res Soc Admin Pharm.

[35] Stewart D, Whittlesea C, Dhital R, Newbould L, McCambridge J (2020). Community pharmacist led medication reviews in the UK: A scoping review of the medicines use review and the new medicine service literatures. Res Soc Admin Pharm.

[36] Medical Research Council (2000). A Framework for Development and Evaluation of RCTs for Complex Interventions to Improve Health In.

[37] Shiell A, Hawe P, Gold L (2008). Complex interventions or complex systems? Implications for health economic evaluation. BMJ.

[38] Petticrew M (2011). When are complex interventions 'complex'? When are simple interventions 'simple'?. Eur J Public Health.

[39] Leventhal H, Brissette I, Leventhal EA, Cameron LD, Leventhal H (2003). The common-sense model of self-regulation of health and illness. The Self-Regulation of Heatlh and Illness Behaviour.

[40] Horne R, Chapman SC, Parham R, Freemantle N, Forbes A, Cooper V (2013). Understanding patients' adherence-related beliefs about medicines prescribed for long-term conditions: a meta-analytic review of the Necessity-Concerns Framework. PLoS ONE.

[41] Rousseau N, Turner KM, Duncan E, O'Cathain A, Croot L, Yardley L, Hoddinott P (2019). Attending to design when developing complex health interventions: a qualitative interview study with intervention developers and associated stakeholders. PLoS ONE.

[42] Gaume J, Grazioli VS, Paroz S, Fortini C, Bertholet N, Daeppen JB (2021). Developing a brief motivational intervention for young adults admitted with alcohol intoxication in the emergency department - Results from an iterative qualitative design. PLoS ONE.

[43] Madden M, Morris S, Stewart D, Atkin K, Gough B, McCambridge J (2019). Conceptualising alcohol consumption in relation to long-term health conditions: Exploring risk in interviewee accounts of drinking and taking medications. PLoS ONE.

[44] Gough B, Madden, M., Morris, S., Atkin, K., McCambridge, J.: How do older people normalise their drinking?: a discursive analysis of interviewee accounts. *Appetite* 2019 (in press). 10.1016/j.appet.2019.104513 31751631

[45] Stewart D, Whittlesea C, Dhital R, Newbould L, McCambridge J (2019). Community pharmacist led medication reviews in the UK: A scoping review of the medicines use review and the new medicine service literatures. Res Soc Admin Pharm.

[46] Madden M, Morris S, Ogden M, Lewis D, Stewart D, O'Carroll RE, McCambridge J (2021). Introducing alcohol as a drug in medicine reviews with pharmacists: findings from a co-design workshop with patients. Drug Alcohol Rev.

[47] Craig P, Dieppe P, Macintyre S, Michie S, Nazareth I, Petticrew M (2008). Developing and evaluating complex interventions: the new Medical Research Council guidance. BMJ.

[48] Cane J, O'Connor D, Michie S (2012). Validation of the theoretical domains framework for use in behaviour change and implementation research. Implement Sci.

[49] Stewart D, van Dongen A, Watson M, Mandefield L, Atkin K, Dhital R, Foster B, Gough B, Hewitt C, Madden M (2020). A pilot cluster randomised trial of the medicines and alcohol consultation (MAC): an intervention to discuss alcohol use in community pharmacy medicine review services. Bmc Health Serv Res.

[50] Stewart D, Madden M, Van Dongen A, Watson M, Morris S, Whittlesea C, McCambridge J (2021). Process study within a pilot cluster randomised trial in community pharmacy: an exploration of pharmacist readiness for research. Res Soc Admin Pharm.

[51] Madden M, Morris S, Ogden M, Lewis D, Stewart D, McCambridge J (2020). Producing co-production: reflections on the development of a complex intervention. Health Expect.

[52] Atkin K, Madden M, Morris S, Gough B, McCambridge J (2021). Community pharmacy and public health: preserving professionalism by extending the pharmacy gaze?. Sociol Health Illn.

[53] The NHS Long Term Plan [https://www.longtermplan.nhs.uk/publication/nhs-long-term-plan/]

[54] O'Cathain A, Croot L, Duncan E, Rousseau N, Sworn K, Turner KM, Yardley L, Hoddinott P (2019). Guidance on how to develop complex interventions to improve health and healthcare. BMJ Open.

[55] Turner KM, Rousseau N, Croot L, Duncan E, Yardley L, O'Cathain A, Hoddinott P (2019). Understanding successful development of complex health and healthcare interventions and its drivers from the perspective of developers and wider stakeholders: an international qualitative interview study. BMJ Open.

